# Supplementary material for: Extreme rainfall events alter the trophic structure in bromeliad tanks across the Neotropics
Source: Nat Commun. 2020 Jun 25;11:3215. doi: 10.1038/s41467-020-17036-4 (PMC7316839; doi:10.1038/s41467-020-17036-4)
Supplement: Supplementary file 1 — Supplementary Information [file 41467_2020_17036_MOESM1_ESM.pdf]

Supplementary material

Supplementary Note 1

To calculate combined correction factor (CCF) for each site (supplementary Table 1), we placed: two buckets in an area without canopy cover (open area), ten containers in an area with canopy cover, and two bromeliads (whose effective surface area had been previously measured with ImageJ) in places with canopy cover. These procedures were performed in rainy days. We then performed the following steps to obtain the CCF for each study area:

forest rainfall (mm) = volume of rain in 10 forest containers / total surface area of 10 containers
open area rainfall (mm) = volume of rain in open buckets / total surface area of buckets
actual catchment area = volume of rain captured by bromeliad / forest rainfall (mm)
canopy effect = forest rainfall (mm) /open area rainfall (mm)
deflection effect = actual catchment area / effective surface area
*Combined Correction Factor = canopy effect * deflection effect*

Supplementary Table 1 | Combined correction factor (CCF) and effective surface area (ESA) for the experimental bromeliad species from each study site.

**Country**	**Site**	**Bromeliad species**	**CCF**	**ESA (cm^2)**
**Argentina**	Las Gamas	*Aechmea distichantha*	0.357	1039.53
**Brazil**	Cardoso I.	*Quesnelia arvensis*	0.088	4773.27
	Macae	*Neoregelia cruenta*	0.260	1218.00
**Colombia**		*Guzmania* spp.	0.361	2297.89
**Costa Rica**	Pitilla	*Guzmania* spp.	0.397	1622.00
**French Guiana**		*Lutheria splendens*	0.380	1060.00
**Puerto Rico**		*Guzmania* spp.	0.387	1110.14

Supplementary Table 2 | Hydrological parameters and their loadings in a principal component analysis (PCA). Percentages indicate the variance explained by each PCA axis. prop.overflow.days = out of the total number of measurements, the proportion of days water depth was ≥ max.depth (i.e., maximum water depth recorded in the time series); prop.driedout.days = out of the total number of measurements, the proportion of days water depth was < 5 mm; cv.depth = coefficient of variation of water depth measurements recorded in the time series.

	PC1	PC2	PC3
**All sites**	65.9%	28.2%	5.9%
prop.overflow.days	0.3705422	0.9280654	0.03732497
prop.driedout.days	-0.6527861	0.2887993	-0.70033222
cv.depth	-0.6607335	0.2351374	0.71284054
**Argentina**	73.0%	21.7%	5.6%
prop.overflow.days	0.4789729	0.875564	-0.06302798
prop.driedout.days	-0.6268841	0.2909051	-0.72276588
cv.depth	-0.6144927	0.3856965	0.68821287
**Brazil (Macae)**	70.0%	27.5%	2.5%
prop.overflow.days	0.3706576	-0.9284092	-0.02587073
prop.driedout.days	-0.6539823	-0.2806717	0.70251726
cv.depth	-0.6594847	-0.2434744	-0.71119632
**Colombia**	65.7%	28.8%	5.5%
prop.overflow.days	0.3653618	-0.9218547	-0.1292082
prop.driedout.days	-0.6440204	-0.3505513	0.6799644
cv.depth	-0.6721224	-0.1652203	-0.7217712
**Costa Rica**	73.9%	24.7%	1.3%
prop.overflow.days	0.4257976	-0.8974795	-0.1150083
prop.driedout.days	-0.6269862	-0.3843026	0.6776428
cv.depth	-0.6523685	-0.21643	-0.7263424
**French Guiana**	72.2%	23.8%	4.02%
prop.overflow.days	0.4662196	-0.8557896	0.224195
prop.driedout.days	-0.5998646	-0.4920738	-0.6308928
cv.depth	-0.6502321	-0.1596479	0.7427724
**Puerto Rico**	81.9%	17.8%	0.28%
prop.overflow.days	0.4919345	-0.8706309	0.001512632
prop.driedout.days	-0.6157696	-0.3467004	0.707549727
cv.depth	-0.6154902	-0.3489995	-0.706661939

Supplementary Table 3 | Macroinvertebrate families composing each functional group and their occurrence (P = presence) in the experimental bromeliads in each site.

		Sites						
**Functional Group**	**Family**	**Argentina**	**Cardoso Island**	**Colombia**	**Costa Rica**	**French Guiana**	**Macaé**	**Puerto Rico**
**Engulfer**	Coenagrionidae		P	P		P	P	
	Corethrellidae		P			P	P	
	Culicidae (*Toxorhynchites* only)				P			P
	Dolichopodidae				P		P	
	Dytiscidae		P			P	P	
	Hydrophilidae	P	P		P		P	
	Periscelididae				P			
	Pseudostigmatidae				P			
	Coleoptera (family undefined)	P						
**Piercer**	Cecidomyiidae		P		P	P		
	Ceratopogonidae (only *Bezzia*, *Sphaeromias*, *Stilobezzia*, *Culicoides*)		P	P		P	P	
	Chironomidae (only Tanypodinae)		P				P	P
	Empididae		P					
	Hydrophilidae		P					
	Lampyridae		P				P	
	Tabanidae	P	P		P	P	P	
**Filter feeder**	Culicidae (except *Toxorhynchites*)	P	P	P	P	P	P	P
**Gatherer**	Aeolosomatidae					P		
	Anisopodidae			P				
	Ceratopogonidae (except *Bezzia*, *Sphaeromias*, *Stilobezzia*, *Culicoides*)		P	P	P		P	
	Chironomidae (except Tanypodinae)	P	P	P	P		P	P
	Enchytraeoidae							P
	Ephydridae		P				P	
	Naididae		P		P	P	P	
	Phoridae		P				P	
	Psychodidae	P	P	P	P		P	
	Scatopsidae			P				
	Sciaridae		P					
	Sphaeroceridae							P
	Stratiomyidae	P	P	P				
	Syrphidae	P	P	P	P		P	
**Scraper**	Candonidae							P
	Elmidae						P	
	Limnocytheridae		P			P	P	P
	Scirtidae	P	P	P	P	P	P	P
	Ephemeroptera (family unknown)		P					
**Shredder**	Calamoceratidae		P					
	Ptilodactylidae			P				
	Tipulidae	P	P	P	P	P	P	P

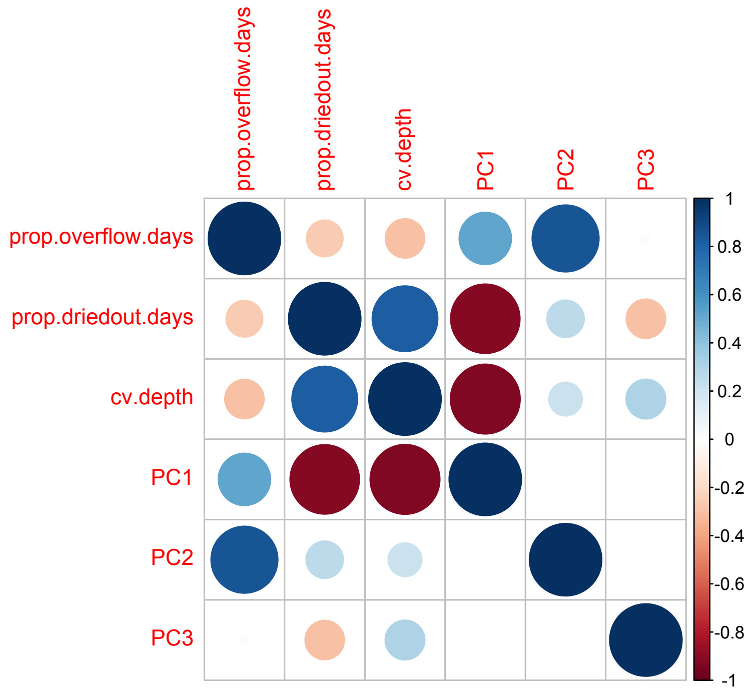

Supplementary Fig. 1 | Correlation plot. Correlations between each hydrological parameter and the first three axes of the Principal Component Analysis (PCA) (all sites). See supplementary Table 2 and Methods for details.

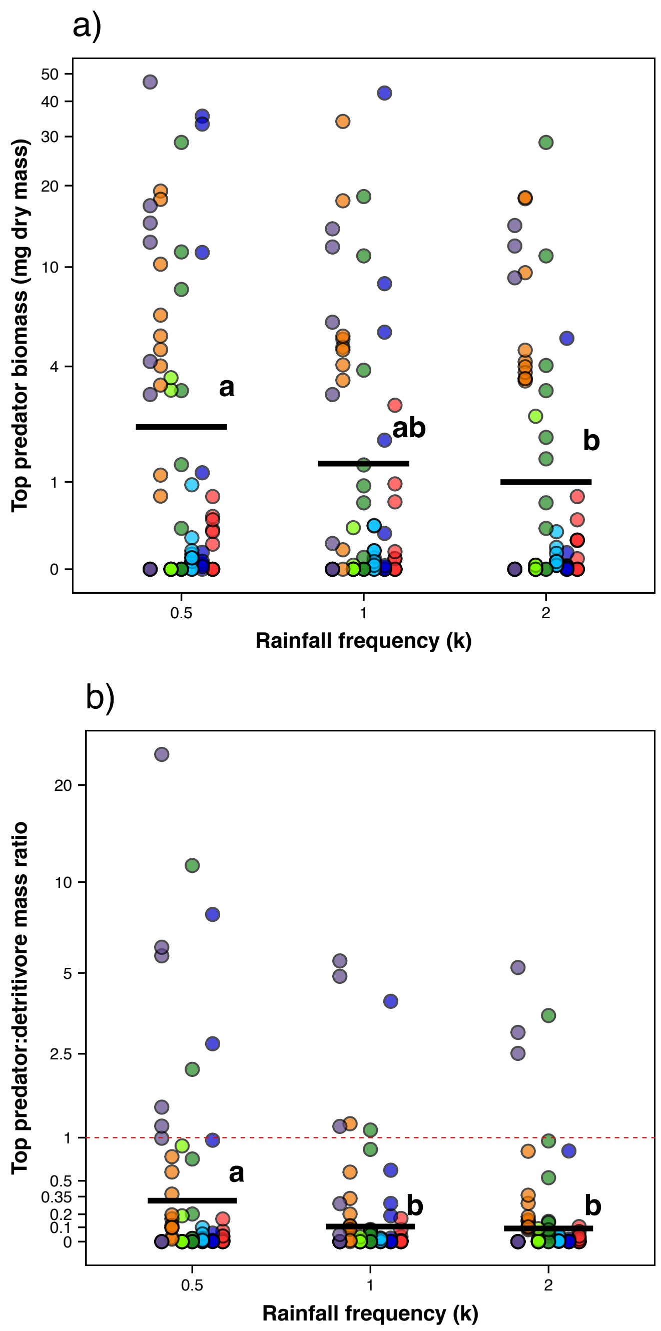

Supplementary Figure 2 | Standing stock of top-predators and pyramid shape change under rainfall frequency. (a)Influence of rainfall frequency (k scalar) on top predator standing stock, and (b) top predator-detritivore mass ratio (pyramid shape). Horizontal bars denote mean values; different letters indicate statistically significant differences (Tukey contrasts, $\alpha = 0.05$). In (a), k of 0.5 and 2 differ statistically (Tukey, $P = 0.020$). (b), PDMR >1 (threshold in red dashed line) indicates bromeliads with inverted trophic pyramids. k equals to 1 represents typical average values of rainfall frequency; values lower and higher than 1 represent deviance from typical values, and characterize extreme events. Circle colors indicate sites of the experiment; colors match with the sites plotted in Fig. 1a. Jitter function was used to add random noise to data in order to prevent overplotting.

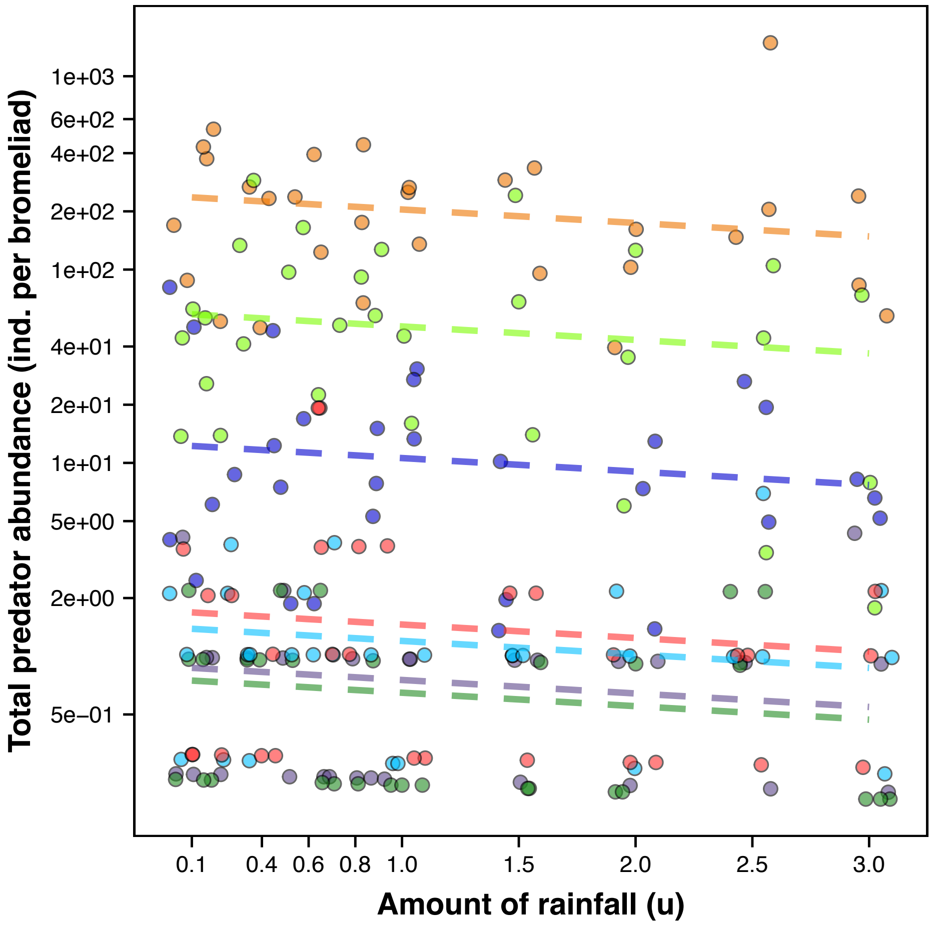

Supplementary Figure 3 | Predator abundance decreases with increasing amount of rainfall (μ). Influence of amount of rainfall on total predator abundance. μ equals to 1 represent typical average values of rainfall frequency; values lower and higher than 1 represent deviance from typical values, and characterize extreme events. Regression lines represent the best fitted negative binomial models predicted using *visreg* function in R. The best model detected site (GLM, $\chi^2 = 2634.0$, $P < 0.001$) and μ (GLM, $\chi^2 = 9.74$, $P=0.002$) influencing predator abundance, with no significant interaction between these variables. Labels on all y-axes represent untransformed values of variables but axes are on logarithm scales. Circle colors indicate sites of the experiment; colors match with the sites plotted in Fig. 1a. Jitter function was used to add random noise to data in order to prevent overplotting.

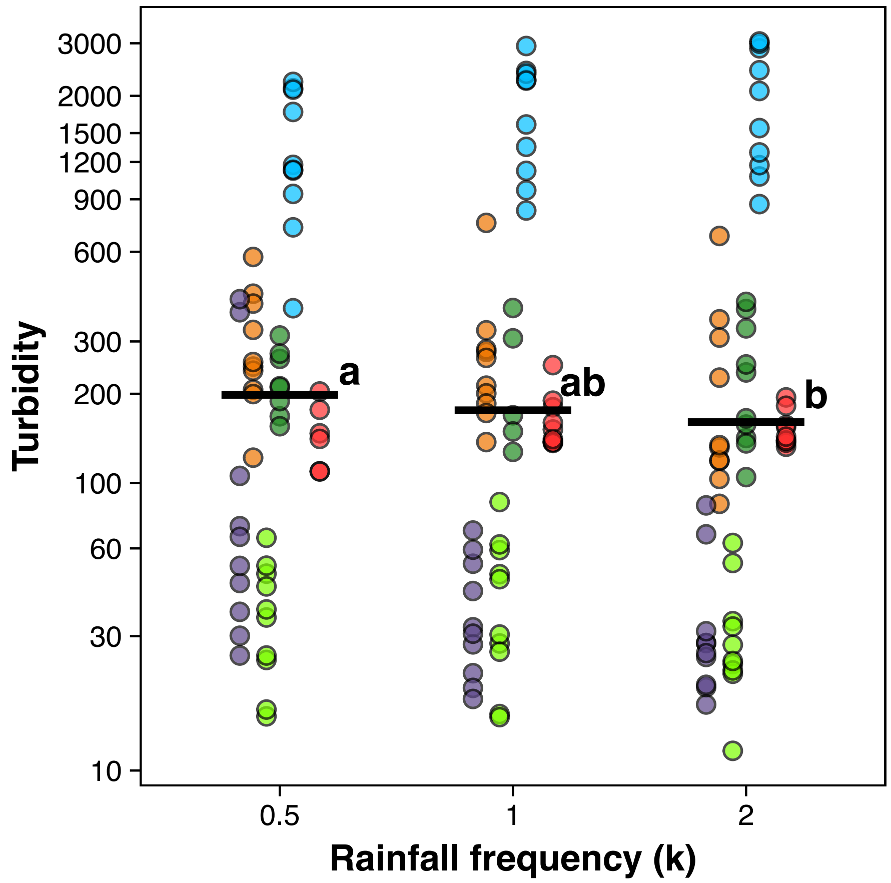

Supplementary Figure 4 | Turbidity changes under rainfall frequency (k). Influence of rainfall frequency (k scalar) on water turbidity, a surrogate for freshwater ecosystem productivity. Horizontal bars denote mean values; different letters indicate statistically significant differences (Tukey contrasts, $\alpha = 0.05$). k of 0.5 and 2 differs statistically (Tukey, P = 0.021). The best-fitted negative binomial model detected a significant site by k scalar interaction (GLM, $\chi^2 = 44.7$, P <0.001), besides of the main effects (GLM, $\chi^2_{site} = 1259.2$, P <0.001; $\chi^2_{site} = 7.4$, P =0.025). However, the Tukey results were averaged over the levels of site, and were performed on the log scale using the function *emmeans* in R, which took into account the interactive model. k equals to 1 represents typical average values of rainfall frequency; values lower and higher than 1 represent deviance from typical values, and characterize extreme events. Circle colors indicate sites of the experiment; colors match with the sites plotted in Fig. 1a. Jitter function was used to add random noise to data in order to prevent overplotting.
