# Supplementary material for: Extreme rainfall events alter the trophic structure in bromeliad tanks across the Neotropics
Source: Nat Commun. 2020 Jun 25;11:3215. doi: 10.1038/s41467-020-17036-4 (PMC7316839; doi:10.1038/s41467-020-17036-4)
Supplement: Supplementary file 3 — Reporting Summary [file 41467_2020_17036_MOESM3_ESM.pdf]

natureresearch

Corresponding author(s): Gustavo Romero

Last updated by author(s): Apr 13, 2020

Reporting Summary

Nature Research wishes to improve the reproducibility of the work that we publish. This form provides structure for consistency and transparency in reporting. For further information on Nature Research policies, seeAuthors & Referees and theEditorial Policy Checklist .

Statistics

For all statistical analyses, confirm that the following items are present in the figure legend, table legend, main text, or Methods section.

n/a	Confirmed	
☐	☒	The exact sample size (*n*) for each experimental group/condition, given as a discrete number and unit of measurement
☐	☒	A statement on whether measurements were taken from distinct samples or whether the same sample was measured repeatedly
☐	☒	The statistical test(s) used AND whether they are one- or two-sided<br>*Only common tests should be described solely by name; describe more complex techniques in the Methods section.*
☐	☒	A description of all covariates tested
☐	☒	A description of any assumptions or corrections, such as tests of normality and adjustment for multiple comparisons
☐	☒	A full description of the statistical parameters including central tendency (e.g. means) or other basic estimates (e.g. regression coefficient) AND variation (e.g. standard deviation) or associated estimates of uncertainty (e.g. confidence intervals)
☐	☒	For null hypothesis testing, the test statistic (e.g. *F*, *t*, *r*) with confidence intervals, effect sizes, degrees of freedom and *P* value noted<br>*Give P values as exact values whenever suitable.*
☒	☐	For Bayesian analysis, information on the choice of priors and Markov chain Monte Carlo settings
☐	☒	For hierarchical and complex designs, identification of the appropriate level for tests and full reporting of outcomes
☒	☐	Estimates of effect sizes (e.g. Cohen's *d*, Pearson's *r*), indicating how they were calculated

Our web collection on statistics for biologists contains articles on many of the points above.

Software and code

Policy information about availability of computer code

Data collection	The database is originated from replicated field experiments. No software was used for data collection.
Data analysis	All the analyses were conducted using the free R software and language. We used R version 3.6.0 (2019-04-26)<br><br>Data availability<br>The data that support the findings of this study are available at http://doi.org/10.5281/zenodo.1124951. This data was collated and hydrologic metrics calculated by a custom-built R package, BWGTools, available at: http://doi.org/10.5281/zenodo.1120418.<br><br>Code availability<br>The R code used to calculate the precipitation treatments is publically archived at http://doi.org/10.5281/zenodo.18548.

For manuscripts utilizing custom algorithms or software that are central to the research but not yet described in published literature, software must be made available to editors/reviewers. We strongly encourage code deposition in a community repository (e.g. GitHub). See the Nature Research guidelines for submitting code & software for further information.

Data

Policy information about availability of data

All manuscripts must include a data availability statement. This statement should provide the following information, where applicable:

- Accession codes, unique identifiers, or web links for publicly available datasets
- A list of figures that have associated raw data
- A description of any restrictions on data availability

The data that support the findings of this study are available at http://doi.org/10.5281/zenodo.1124951. This data was collated and hydrologic metrics calculated by a custom-built R package, BWGTools, available at: http://doi.org/10.5281/zenodo.1120418. The R code used to calculate the precipitation treatments and analyse

data are publically archived at, respectively: http://doi.org/10.5281/zenodo.18548 and http://doi.org/10.5281/zenodo.1124949.

Field-specific reporting

Please select the one below that is the best fit for your research. If you are not sure, read the appropriate sections before making your selection.

☐ Life sciences ☐ Behavioural & social sciences ☒ Ecological, evolutionary & environmental sciences

For a reference copy of the document with all sections, see nature.com/documents/nr-reporting-summary-flat.pdf

Ecological, evolutionary & environmental sciences study design

All studies must disclose on these points even when the disclosure is negative.

Study description	This is a multi-site coordinated experiments to show how variation in the quantity and evenness of rainfall modulates trophic structure in 210 natural freshwater ecosystems (tank bromeliads) across Central and South America (18°N to 29°S).
Research sample	We used natural, detritus-based microcosms (bromeliad phytotelmata) as model systems due to their widespread distribution and ease of manipulation. Bromeliad aquatic ecosystems are inhabited by a diverse fauna of macroinvertebrates (insects, crustaceans), comprising top predators, mesopredators, and detritivores.
Sampling strategy	At the end of the experiment (60th day), we dissected each bromeliad by removing and washing each leaf separately in running water and then filtered this water through 125 and 850 µm sieves. We recorded the morphospecies and abundance of all aquatic macroinvertebrates (body size larger than 0.5 mm). We determined the body size and trophic position of each individual organism surveyed. Trophic position was determined from our own feeding trials, gut contents and from the literature. To calculate invertebrate body mass, we used allometric equations between the body length and dry mass, or mean of dry mass for very small insects.<br>For each experimental site, we used 30 bromeliads (i.e., 30 sample units), which were randomly selected to receive one of the treatments described in the item "Reproducibility" below. Sample size (n=30) is predefined because of the combinations of treatments (10 levels of treatment µ and 3 levels of the treatment k)
Data collection	Data collection is described above and in M&Ms.<br>Below there is a list of researchers that collected data in each site:<br>Las Gamas (Argentina):<br>Ignacio Brberis, Rodrigo Freire, Guillermo Montero<br>Cardoso (Brazil):<br>Gustavo Romero, Pablo Antiqueira, Gustavo C Piccoli<br>Macae (Brazil):<br>Vinicius F Farjalla, Nicholas Marino, Alice Campos, Juliana Leal, Andrew McDonald<br>Colombia:<br>Fabiola Ospina, Emilio Realpe, M. Kurtis Trzcinski<br>Pitilla (Costa Rica):<br>Diane Srivastava, Sarah Amundrud, M. Kurtis Trzcinski<br>French Guiana:<br>Regis Cereghino, Celine Leroy, Bruno Corbara, Jean-François Carrias<br>Puerto Rico:<br>Dimaris Mercado, Regis Cereghino, M. Kurtis Trzcinski
Timing and spatial scale	The field experiments lasted 60 days. We replicated the experiment in seven sites across the Neotropics. Within each site, the 30 experimental bromeliads were at least 2 m apart each other.
Data exclusions	No data were excluded from the analyses.
Reproducibility	We contrasted rainfall-mediated changes in hydrological stability of the study system with the effects of two main rainfall components: (i) the mean daily amount of rainfall, µ; and (ii) distribution of rainfall events around this mean through time, k (i.e., a measure of rainfall frequency). Sites with many dry days and infrequent rainfall resulted in low µ and low k respectively. Ambient levels of the rainfall components were first determined using recent meteorological data from each site (see Methods). We applied a negative binomial distribution to these data to estimate the parameters µ and k. We randomly applied ten levels of µ (ranging from 0.1 to 3.0) and three levels of k ranging from 0.5 to 2.0 in a fully factorial experimental design at each of our seven sites for a total of $10 \times 3 \times 7 = 210$ food webs in individual bromeliads. This allowed us to compare ambient, baseline conditions ($\mu = 1$, $k = 1$) and extreme fluctuations of rainfall quantity (10-300%) and frequency (50-200%) to average historical levels of daily variability for each site.<br>Each of 210 experimental bromeliads (30 bromeliads x 7 sites) represented an independent sample unit. The experiment was replicated in seven distinct sites. All attempts at replication were successful.
Randomization	In each site, we randomly selected thirty bromeliads of the most abundant species and with the most common size. We used bromeliads that had more than 100 ml of tank capacity and thus can be colonized by the large predators. To remove any residual invertebrates, we hung the bromeliads upside down, and let them dry for seven days. For each site we homogenized detritus and

invertebrates collected from these bromeliads, then used equal aliquots of the mixture to initiate the community assembly in the experimental ecosystems27. We employed individual transparent plastic shelters above each bromeliad to prevent natural rainfall into the plants.

Blinding

Each experiment was replicated seven times across the Neotropics. The PIs and their research groups conducted the experiment in these different sites following a standardized protocol. Typically the researchers cannot see what organisms are living into the bromeliad wells before dessicating them in the laboratory.

Did the study involve field work? [x] Yes [ ] No

Field work, collection and transport

Field conditions

Each field site presented particular environmental conditions. These variations were controlled in our experiments. All the 7 replicated experiments were conducted during the rainy season.

Location

We replicated the experiment at seven sites across Central and South America (from 29°S to 18°N), including Las Gamas (Argentina), Cardoso and Macae (Brazil), Colombia, Pitilla (Costa Rica), French Guiana, and Puerto Rico.
Below we present latitude and longitude, in degrees:
Las Gamas (Argentina): 29.39 S, 60.33W
Cardoso (Brazil): 25.07 S, 47.92 W
Macae (Brazil): 22.38 S, 41.75 W
Colombia: 5.07 N, 75.45 W
Pitilla (Costa Rica): 10.98 N, 85.43 W
French Guiana: 5.06 N, 53.05 W
Puerto Rico: 18.30 N, 65.79 W

Access and import/export

All the experiments were conducted following the rules and laws of the related coutries. No material was necessary to import or export. All the materials were analysed in local laboratories, following local rules and laws. When necessary (depending on local rules), permits for conducting the experiments were requested and approved.

Disturbance

No significant disturbance was caused by manipulating small freshwater ecosystems

Reporting for specific materials, systems and methods

We require information from authors about some types of materials, experimental systems and methods used in many studies. Here, indicate whether each material, system or method listed is relevant to your study. If you are not sure if a list item applies to your research, read the appropriate section before selecting a response.

Materials & experimental systems

n/a	Involved in the study
[x]	[ ] Antibodies
[x]	[ ] Eukaryotic cell lines
[x]	[ ] Palaeontology
[ ]	[x] Animals and other organisms
[x]	[ ] Human research participants
[x]	[ ] Clinical data

Methods

n/a	Involved in the study
[x]	[ ] ChIP-seq
[x]	[ ] Flow cytometry
[x]	[ ] MRI-based neuroimaging

Animals and other organisms

Policy information about studies involving animals; ARRIVE guidelines recommended for reporting animal research

Laboratory animals

The study did not involve laboratory animals

Wild animals

While the taxonomic compositions of macroinvertebrate communities are site specific, all taxa can be assigned to three trophic levels: detritivores, mesopredators, and top-predators24,25. The detritivores are typically represented by larvae of Diptera (Chironomidae (except Tanypodinae), Culicidae (except Toxorhynchites), Syrphidae, Tipulidae) and Coleoptera (Scirtidae). The largest top predators are represented by larvae of damselfly (Coenagrionidae), horsefly (Tabanidae), adult Coleoptera (Dytiscidae), and leeches (Hirudinea). The mesopredators often include larvae of Ceratopogonidae (Bezzia spp.), Corethrellidae, Toxorhynchites and Tanypodinae.

Field-collected samples

In each site, we selected thirty bromeliads of the most abundant species and with the most common size. We used bromeliads that had more than 100 ml of tank capacity and thus can be colonized by the large predators. We washed each bromeliad with spring water to remove detritus and organisms. To remove any residual invertebrates, we hung the bromeliads upside down, and let them dry for seven days. For each site we homogenized detritus and invertebrates collected from these bromeliads, then used equal aliquots of the mixture to initiate the community assembly in the experimental ecosystems. We employed individual transparent plastic shelters above each bromeliad to prevent natural rainfall into the plants. The rain shelters were settled high

enough to ensure that they did not alter macroinvertebrate colonization or temperature within the bromeliads. We randomly divided the 30 treatment combinations into three blocks of ten bromeliads, and initiated each block on one of three consecutive days. This procedure also allowed enough time to sample invertebrates at the end of the experiment.

In order to estimate the key hydrological parameters, we measured water depth in the central and two lateral leaf wells of each bromeliad every two days, and used average values per bromeliad. The hydrological parameters for each bromeliad included: (i) coefficient of variation of water depth across the entire experiment, (ii) proportion of overflow days, i.e., the number of days water depth was ≥ maximum depth recorded divided by the total number of measurements, (iii) proportion of dried-out days, i.e., the number of days water depth was < 5 mm divided by the total number of measurements. These hydrological parameters were used to create a metric of hydrological stability, using Principal Component Analysis (PCA). We used the scores of the first axis of the PCA (supplementary Table 2), for each site, to summarize these parameters into a single variable of hydrological stability. This axis quantified a gradient of habitat permanence and stability, where increasing scores represent more stable ecosystems (i.e., ecosystems that dried out less often, and held more water throughout the experiment, supplementary Table 2).

At the end of the experiment (60th day), we recorded with hand-held data loggers water turbidity, a measure that integrates organic and inorganic suspended matter, including free-living algae and particulate nutrients (C, N, P), resulting from detritivore activity. Thus, turbidity represents a surrogate for total nutrient availability in freshwater ecosystems. Then, we dissected each bromeliad by removing and washing each leaf separately in running water and then filtered this water through 125 and 850 µm sieves. We recorded the morphospecies and abundance of all aquatic macroinvertebrates (body size larger than 0.5 mm). We recorded 4 to 38 morphospecies per bromeliad (mean per bromeliad ± SD: 14.7 ± 9.4). We determined the body size and trophic position of each individual organism surveyed. Trophic position was determined from our own feeding trials, gut contents, stable isotope analyses and from the literature. To calculate invertebrate body mass, we used allometric equations between the body length and dry mass, or mean of dry mass for very small insects.

Ethics oversight

No ethical approval or guidance was required because the experiment involved only invertebrates. And the countries were we conducted the experiments apparently have no specific laws that limit invertebrate manipulations.

Note that full information on the approval of the study protocol must also be provided in the manuscript.
